# The impact of asymptomatic ventricular arrhythmias on the outcome of heart failure patients with reduced ejection fraction

**DOI:** 10.1186/s43044-022-00247-z

**Published:** 2022-02-16

**Authors:** Mohamed Sanhoury, Fatema Mohamed, Mohamed Sadaka, Mohamed Ayman Abdel-Hay, Mohamed Sobhy, Mostafa Elwany

**Affiliations:** Cardiology and Angiology Department, Faculty of Medicine, University of Alexandria, Champollion Street, Al Mesallah Sharq, 21526 Alexandria Governorate Egypt

**Keywords:** Heart failure, Ventricular arrhythmias, Prognosis

## Abstract

**Background:**

Ventricular arrhythmias cause a significant proportion of sudden deaths. Several studies demonstrate a high prevalence of ventricular arrhythmias in patients with heart failure regardless of the etiology. The aim of this study was to determine the prevalence of silent ventricular arrhythmias in ambulatory heart failure patients with reduced left ventricular ejection fraction (HFrEF) and its correlation to the prognosis.

**Results:**

Four hundred (400) ambulatory HFrEF patients on maximum tolerated doses of heart failure medications were included. Holter monitoring for 7 days was done in all patients searching for silent ventricular arrhythmias. The patients were followed-up for one year to detect the occurrence of major adverse cardiovascular events. We divided the study population into 2 groups based on an LVEF cutoff value of 30% (Group A < 30%, Group B ≥ 30%). Holter monitoring revealed ventricular arrhythmias in 304 patients. Patients with left ventricular ejection fraction (EF) < 30% (Group A) had more complex ventricular arrhythmias in the form of frequent Premature ventricular contractions (PVCs) of ≥ 5% and or non-sustained ventricular tachycardia (NSVT) runs. Furthermore, Among Group A, more major cardiovascular events were observed. Multivariate regression analysis showed that frequent PVCs and severely reduced LVEF were the strongest independent predictors of major cardiovascular events.

**Conclusions:**

ventricular arrhythmias are common in HFrEF patients even in the compensated status. Both, left ventricular systolic function and the PVCs burden were found to be the strongest predictors of major adverse cardiovascular events.

## Background

Heart failure is a clinical syndrome characterized by typical symptoms (fatigue and shortness of breath) that could be associated by clinical signs of systemic or pulmonary venous congestion or both. This is caused by cardiac structural and/or functional abnormality. HF has been divided into three phenotypes based on the of left ventricular ejection fraction (LVEF); heart failure with reduced ejection fraction (HFrEF) with the EF ≤ 40%, heart failure with mildly reduced ejection fraction (HFmrEF) where the EF is from 41–49% and heart failure with preserved ejection fraction (HFpEF), with the EF ≥ 50% [1].

Premature ventricular complexes (PVCs) are the most common ventricular arrhythmias. Their impact on the patient's prognosis depends on the presence of cardiac structural and/or functional abnormalities. In the absence of an underlying structural heart disease, PVCs are regarded as a benign phenomenon [2].

Several studies have reported that frequent PVCs could be a trigger for ventricular tachycardia (VT), ventricular fibrillation (VF) and sudden cardiac death (SCD). Moreover, frequent PVCs have been found to cause not only symptoms like palpitations, chest discomfort, and syncope but also contribute to adverse cardiac remodeling and the risk of developing new onset or worsening of heart failure (HF) [3, 4].

Several recent therapeutic approaches, whether pharmacological or device-based, have been introduced into clinical practice to improve the clinical outcome of HF patients. However, the high rate of re‐hospitalization and high cardiac mortality are still serious issues [5].

Based on data from a number of studies, almost 80% of patients with reduced left ventricular systolic function have frequent ventricular premature contractions (PVCs), whereas > 40% have runs of non-sustained ventricular tachycardia (NSVT) [6]. Sustained ventricular tachycardia, frequent PVCs or NSVT were found to be predictors of total mortality and morbidity in a number of studies. These arrhythmias are especially common in those with ischemic etiology and those with a lower left ventricular ejection fraction (LVEF) [7, 8].

The aim of the present study was to determine the prevalence of silent ventricular arrhythmias on one-week ambulatory ECG monitoring in clinically compensated outpatient heart failure patients with reduced ejection fraction (HFrEF) and its impact on the prognosis (death, HF hospitalization and sustained VT).

## Methods

### Study population

The study included 400 compensated heart failure patients with reduced ejection fraction (EF 25–40%) who follow regularly at the outpatient heart failure clinic of Alexandria Main University Hospital and receiving the maximal tolerated doses of beta-blockers, angiotensin converting enzyme inhibitors/Angiotensin II receptor blockers (ACEI/ARBs) and mineralocorticoid receptor antagonists (MRA).

Due to financial constraints, no patients were taking angiotensin receptor-neprilysin inhibitors (ARNI).

All participants were informed about the study and signed a written consent regarding this study. This study complied with the declaration of Helsinki and was reviewed and approved by the Ethical committee of the Faculty of Medicine, Alexandria University (Review report serial number 0302694). The diagnosis of HF was determined according to the current HF guidelines [1].

### We excluded the following patients

Patients older than 70 years, more than mild anemia (< 11 g/dl), evidence of sustained or non-sustained VT on surface ECG, prior cardiac arrest, active metabolic abnormalities (severe renal impairment with eGFR ≤ 30 ml/min/1.73 m^2^, abnormal TSH, electrolyte disturbances), current active ischemia (ongoing chest pain, new ECG changes), those with cardiac resynchronization therapy (CRT) implantation or treated with class III antiarrhythmic drugs.

### Methods

The baseline visit included full clinical assessment, 12 lead resting ECG, routine lab investigations followed by one week ambulatory Holter monitoring (3 Channel 5 Lead Mortara H3+, Preventice Solutions, Inc.), to detect silent ventricular arrhythmias (analyzed by 2 electrophysiologists). During Holter monitoring, patients were instructed to do their usual daily activities.

Clinical follow up (including 12 lead resting ECG) was performed at 1, 3, 6 and 12 months intervals. Occurrence of any of the major adverse cardiovascular events (death, HF hospitalization or sustained VT) was recorded.

### Statistical analysis

Data were fed to the computer and analyzed using IBM SPSS software package version 20.0. (Armonk, NY: IBM Corp). Qualitative data were described using number and percent. Quantitative data were described using range (minimum and maximum), mean, standard deviation, median and interquartile range (IQR). Significance of the obtained results was judged at the 5% level.

## Results

Patients included in this study were predominantly males (93%) with only 7% were females. Their age ranged from 40 to 69 years. The LV ejection fraction ranged from 25 to 40% with mean value of 32.33 ± 4.88. Forty eight percent of the patients had severely reduced LV systolic function with EF < 30% (Group A).

### ECG parameters

The heart rate ranged from 55 to 100 bpm (mean heart rate was 78 bpm), the PR interval ranged from 120 to 240 ms with 36 patients (9%) suffered from first degree heart block, 79 patients (19%) had left bundle branch block (LBBB) and 32 patients (8%) had right bundle branch block (RBBB).

### Coronary angiography

Was done in all study patients (as a part of the study protocol) to rule out the presence of significant coronary artery disease, 328 patients (82%) had CAD whether single or multivessel disease.

### 2D echocardiography

LV systolic function was measured by Simpson-based assessment of LVEF. It was observed that 48% of the patients had severely reduced LV systolic function of < 30% (Group A) and the remaining 52% of the patients had an ejection fraction which ranged from 30 to 39% (Group B).

### Holter monitoring (Table 1)

**Table 1:** Shows the distribution of the studied cases according to 1-week Holter data

Heart rate (bpm)	54–120 (72 ± 10)
Supraventricular ectopics	144 patients
SVT	40 patients
PVCs	
Infrequent < 5%	108 patients
Frequent ≥ 5%	196 patients

bpm: beats per minute, SVT: supraventricular tachycardia, PVCs: premature ventricular contractions

- Heart rate ranged from 54 to 120 bpm.
- Supraventricular premature contractions were seen in 144 (36%) patients, 40 (10%) patients had short runs of supraventricular tachycardia (SVT) with heart rate reaching up to 170 followed by spontaneous termination.
- PVCs were recorded in 304 (76%) patients, 108 (27%) patients had infrequent PVCs. 196 (49%) patients had frequent PVCs. All PVCs were monomorphic with no sustained VT recorded on Holter monitoring.
- No episodes of paroxysmal AF were recorded in any of the study patients.

### Distribution of PVC burden in both cardiomyopathy groups (Table 2)

**Table 2:** Shows the distribution of premature ventricular contraction burden in both cardiomyopathy groups

	Cardiomyopathy			
	Ischemic (*n* 236)		Non-ischemic (*n* 68)	
	No.	%	No.	%
Infrequent PVCs (< 5%)	84	35.6	24	35.3
Frequent (≥ 5%)	152	64.4	44	64.7

PVCs: premature ventricular contractions

Ischemic cardiomyopathy (ICM) group including 236 patients: infrequent PVCs were detected in 84 patients (35.6%) while frequent PVCs were detected in 152 patients (64.4%).

Non-ischemic cardiomyopathy (NICM) group including 68 patients: infrequent PVCs were detected in 24 patients (35.3%) while 44 patients (64.7%) had frequent PVCs.

**Fig. 1:**
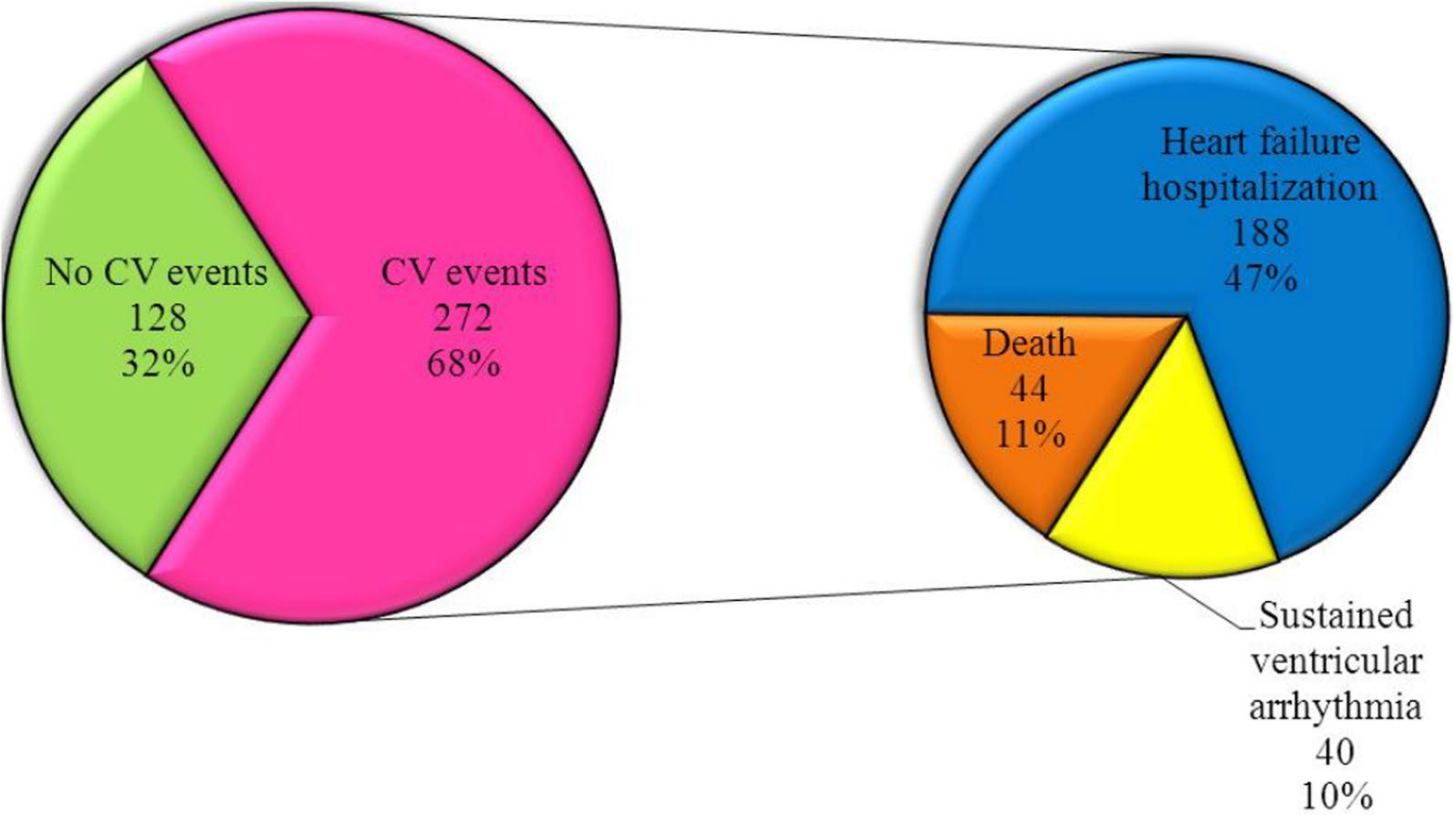
Distribution of the studied cases according to occurrence of major cardiovascular events (*n* = 400)

During the follow up period (12 months), major cardiovascular events were encountered in 272 patients (68%), among which 44 patients (11%) died, 188 patients (47%) suffered from worsening of heart failure symptoms requiring hospitalization and 40 patients (10%) experienced sustained VT with hospital admission. (Table 3 and Fig. 1).

**Table 3:** Distribution of the studied cases according to occurrence of major cardiovascular events (*n* = 400)

Major cardiovascular events	No.	%
No CV events	128	32.0
CV events	272	68.0
Death	44	11.0
Heart failure hospitalization	188	47.0
Sustained ventricular arrhythmia	40	10.0

CV: cardiovascular

Table 4 shows the prevalence of PVCs based on severity LV dysfunction in our patient population.

**Table 4:** Prevalence of premature ventricular contractions on Holter monitor based on the severity of left ventricular dysfunction (*n* = 304)

LVEF	PVC burden for 1 week Hotler			
	Infrequent (< 5%) (*n* = 108)		Frequent (≥ 5% or NSVT) (*n* = 196)	
	No	%	No	%
Severely reduced LVEF < 30 (Group A)	56	51.9	112	57.1
Reduced LVEF ≥ 30 (Group B)	52	48.1	84	42.9

PVC: premature ventricular contractions, LVEF: left ventricular ejection fraction

### Incidence of major cardiovascular events in both cardiomyopathy groups

In ICM group, 40 patients (18.5%) died, 152 patients (70.4%) suffered from worsening of heart failure symptoms with hospital admission while 24 patients (11.1%) experienced sustained ventricular tachycardia with hospital admission.

Among the NICM group, 4 patients died (7.1%) while 36 patients (64.3%) had decompensated heart failure which required hospitalization and 16 patients (28.6%) experienced sustained ventricular tachycardia.

**Fig. 2:**
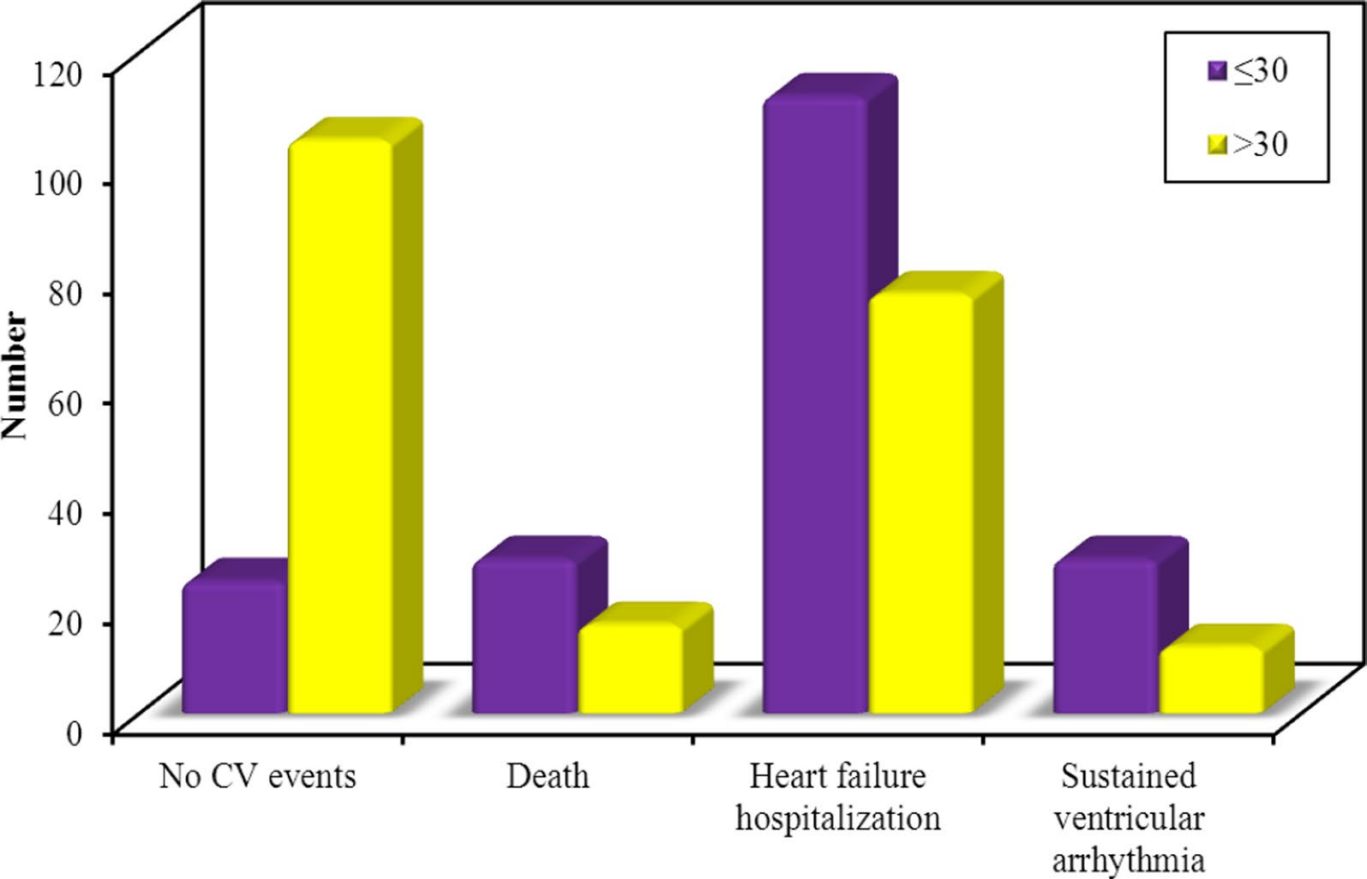
Incidence of major adverse cardiovascular events according to severity of left ventricular dysfunction

There was a statistically significant higher incidence of major cardiovascular events in group A (those with LV ejection fraction of < 30%) (*P* value < 0.001). (Table 5 and Fig. 2). There was also a statistically significant higher incidence of major cardiovascular events in those with high PVC burden in 1 week-Holter monitoring (*P* value < 0.001). (Table 6).

**Table 5:** Shows the association between left ventricular ejection fraction and occurrence of major cardiovascular events

	LVEF		*χ*^2^	*P*	OR	CI. 95% (LL–UL)
	Group A < 30 (*n* = 192)	Group B ≥ 30 (*n* = 208)				
No CV events®	24	104	–	1.000	–	–
Death	28	16	4.843*	0.028*	0.132*	0.062–0.281
Heart failure hospitalization	112	76	19.039*	< 0.001*	0.157*	0.092–0.266
Sustained ventricular arrhythmia	28	12	8.618*	0.003*	0.099*	0.044–0.222
Total CV events	168	104	64.522*	< 0.001*	4.333*	0.086–0.237

*χ*^2^: Chi square test, *P*: P value for association between different categories, OR: Odds ratio, ®: reference group, CI: Confidence interval, LL: Lower limit, UL: Upper Limit, LVEF: left ventricular ejection fraction, CV: cardiovascular

*Statistically significant at *P* ≤ 0.05

**Table 6:** Shows the association between premature ventricular contractions burden on Holter monitoring and occurrence of major cardiovascular events

	PVC burden		*χ*^2^	*P*	OR	CI. 95% (LL–UL)
	Infrequent® (*n* = 108)	Frequent (*n* = 196)				
No CV events ®	48	32	–	1.000	1.000	–
Death	12	28	0.614	0.002*	3.500*	1.55–7.87
Heart failure hospitalization	40	104	7.172*	0.007*	3.900*	2.19–6.94
Sustained ventricular arrhythmia	8	32	4.848*	0.028*	6.000*	2.45–14.67
Total CV events	60	164	28.391*	< 0.001*	0.667*	2.39–7.009

χ^2^: Chi square test, *P*: P value, OR: Odds ratio, ®: reference group CI: Confidence interval, LL: Lower limit, UL: Upper Limit, PVCs: premature ventricular contractions, CV: cardiovascular

*Statistically significant

**Fig. 3:**
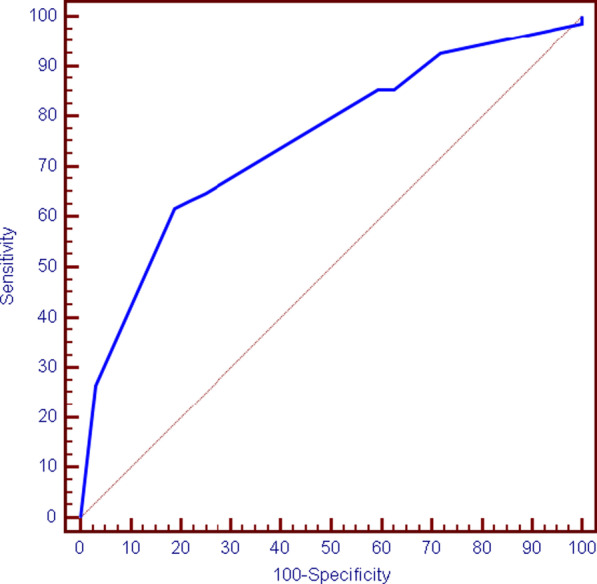
ROC curve for left ventricular ejection fraction to predict total cardiovascular events (*n* = 400)

Multivariate regression analysis of patient characteristics that affect occurrence of major cardiovascular events showed that frequent PVCs (≥ 5% or NSVT) and severely reduced LVEF (< 30%) are the strongest independent factors that affect major cardiovascular events (Fig. 3 and Table 7).

**Table 7:** Agreement (sensitivity, specificity) for left ventricular ejection fraction to predict total cardiovascular events (*n* = 400)

	AUC	*P*	95% C.I	Cut off	Sensitivity	Specificity	PPV	NPV
LVEF	0.749*	< 0.001*	0.652–0.830	≤ 30	61.76	81.25	87.5	50.0

AUC: area under a curve, *P* value: probability value, CI: confidence intervals, NPV: negative predictive value, PPV: positive predictive value

*Statistically significant at *P* ≤ 0.05

## Discussion

Ventricular arrhythmias are common in patients with advanced heart failure, with a prevalence of up to 33% in chronic ambulatory patients and it predict increased mortality from HF [9]. pre-operative ventricular arrhythmias were noted in Up to 45% in patients supported with a left ventricular assist device (LVAD) [10].

The aim of the current study was to determine the prevalence of silent VAs in ambulatory clinically stable HFrEF patients and its correlation to the outcome (death, HF hospitalization and sustained VT).

This study revealed that PVCs were detected during ambulatory ECG monitoring in 304 patients (76%), among which, 108 patients (27%) had infrequent PVCs (less than 5% of the total beats) and 196 patients (49%) had frequent PVCs (5% or more of the total beats). Among the frequent PVCs group, 94 patients (47.95%) had a single or multiple runs of NSVT. Sustained VT was not recorded in any of the studied patients during the ambulatory ECG monitoring period.

Regarding the relation of PVC frequency and the underlying substrate, we observed that among the ICM group (the great majority of our study patients), infrequent PVCs were detected in 84 patients (35.6%) while frequent PVCs in 152 patients (64.4%). Among the NICM group, infrequent PVCs were observed in 24 patients (35.3%), while 44 patients (64.7%) had frequent PVCs. PVCs were common finding in HFrEF patients regardless the etiology.

Podrid et al. [11] conducted a systematic review which included 13 studies. The overall number of patients included were 1322 patients. The authors showed that PVCs are particularly frequent in those who had CHF regardless the etiology (i.e., ischemic vs non-ischemic causes). Almost 80% of CHF patients had frequent PVCs whereas more than 40% had runs of NSVT.

In our study, we observed that in patients (Group A) with severely reduced LV systolic function (EF < 30%), frequent PVCs were detected in 28 patients (57.1%). While among patients with LVEF ≥ 30% (Group B), frequent PVCs were detected in 21 patients (42.9%).

Boas et al. [12], examined both the prevalence and prognostic significance of ventricular arrhythmias in 850 non-ischemic systolic HF patients with LVEF ≤ 35% and elevated natriuretic peptides. They performed a 24-h Holter monitor for all patients looking for NSVT, low PVCs burden (< 30 per hour) and high PVCs burden (≥ 30 per hour). In total, 193 patients died, 49 from sudden cardiac death (SCD) and 125 from cardiovascular death (CVD). NSVT, observed in 365 patients, was significantly associated with increased all-cause mortality. High PVC burden, found in 352 patients, was associated with increased all-cause mortality. There was no statistically significant association with SCD for neither NSVT nor PVC. In interaction analyses, neither NSVT (P = 0.56) nor high burden of PVC (P = 0.97) was associated with survival benefit from ICD implantation.

Our study showed a statistically significant association between the occurrence of major cardiovascular events (total mortality, hospitalization by HF and sustained VT) and the PVCs burden on Holter monitoring (*P* value = 0.008).

Saito [13] et al. examined the factors that predict ventricular arrhythmias (VAs) in the late phase (≥ 7 days) after acute myocardial infarction (AMI). They included 136 consecutive patients with an LVEF of ≤ 40% after AMI. The average LVEF at admission was 32.7 ± 8.2%. During a mean follow-up period of 20.7 months, 14 patients (10%) experienced lethal VAs, including VF (*n* = 8) and sustained VT (*n* = 10). Both age and the admission LVEF predicted lethal VAs on univariate analyses. Receiver operating characteristic curve analysis showed a LVEF cut-off value of 23% predicted the primary endpoint (area under the curve: 0.77, *P* < 0.0001). Furthermore, LVEF at admission independently predicted the primary endpoint (risk ratio = 7.12, *P* = 0.001) on multivariable analysis.

In contrast, other studies had reported no such relation between VAs and SCD. In one report involving 77 patients with CHF, the presence of NSVT on ambulatory monitoring was more common in those with the greatest degree of LV dysfunction and symptomatic CHF [14]. However, NSVT was not independently associated with SCD.

Teerlink et al. [15] examined the independent predictive value of VAs for SCD and all-cause mortality in PROMISE (Prospective Randomized Milrinone Survival Evaluation) trial. They included 1080 patients (NYHA class III/IV) and the LVEF < 35%. VAs were analyzed and quantified by the use of a baseline ambulatory ECGs. The frequency of NSVT was the most powerful predictor and remained a significant independent predictor when included with other clinical variables in the multivariate models of both sudden death mortality and non–sudden death mortality.

Lip et al. had published an interesting paper on arrhythmias in HF. They reported that VAs were frequently seen as the cause of sudden death or resuscitated sudden death in CHF. Indeed, 50% of all deaths in advanced CHF were sudden and the assumption had been that a significant proportion of these were due to VT/VF. As the severity of CHF increases, the percentage of deaths described as sudden decreases although the absolute risk of VAs and sudden death probably continues to increase [16].

In the current study, a multivariate analysis included the following variables (Age, PR interval, QRS duration, bundle branch pattern, LVEF and PVC burden), revealed that LVEF and the PVCS burden were independent predictors of major adverse cardiovascular events (death, HF hospitalization and sustained VT).

We strongly believe that a lot of research is still needed to understand the predictors of prognosis in heart failure (HF) patients, especially the compensated clinically stable patients, and to better stratify them in order to improve the high morbidity and mortality rates. Our study highlighted the importance of through assessment of such a cohort of asymptomatic HFrEF patients searching for silent arrhythmias.

### Limitations

The number of patients enrolled in this study represented a limitation together with the predominance of the male gender and the relatively short follow-up duration.

## Conclusions

Among heart failure patients with reduced ejection fraction, ventricular arrhythmias are common. Both LV systolic function and the burden of PVCs are regarded as the strongest predictors of major adverse cardiovascular events.

## Data Availability

All data analyzed during this research are included in this published article.

## Abbreviations

CAD: Coronary artery disease
CHF: Congestive heart failure
NICM: Non-ischemic cardiomyopathy
HF: Heart failure
HFrEF: Heart failure with reduced ejection fraction
ICM: Ischemic cardiomyopathy
ICD: Implantable cardioverter defibrillator
LBBB: Left bundle branch block
LVEF: Left ventricular ejection fraction
MACE: Major adverse cardiovascular events
NSVT: Non-sustained ventricular tachycardia
PVCs: Premature ventricular contractions,
RBBB: Right bundle branch block
SCD: Sudden cardiac death
SVT: Supraventricular tachycardia
VT: Ventricular tachycardia

## References

[1] McDonagh TA, Metra M, Adamo M, Gardner RS, Baumbach A, Böhm M (2021). 2021 ESC Guidelines for the diagnosis and treatment of acute and chronic heart failure. Eur Heart J.

[2] Kennedy HL, Whitlock JA, Sprague MK, Kennedy LJ, Buckingham TA, Goldberg RJ (1985). Long-term follow-up of asymptomatic healthy subjects with frequent and complex ventricular ectopy. N Engl J Med.

[3] Simpson RJ, Cascio WE, Schreiner PJ, Crow RS, Rautaharju PM, Heiss G (2002). Prevalence of premature ventricular contractions in a population of African American and white men and women: the Atherosclerosis Risk in Communities (ARIC) study. Am Heart J.

[4] Lee GK, Klarich KW, Grogan M, Cha YM (2012). Premature ventricular contraction-induced cardiomyopathy: a treatable condition. Circ Arrhythm Electrophysiol.

[5] Messineo FC (1989). Ventricular ectopic activity: prevalence and risk. Am J Cardiol.

[6] Ponikowski P, Voors AA, Anker SD, Bueno H, Cleland JGF, Coats AJS (2016). ESC Scientific Document Group. 2016 ESC Guidelines for the diagnosis and treatment of acute and chronic heart failure: the Task Force for the diagnosis and treatment of acute and chronic heart failure of the European Society of Cardiology (ESC) Developed with the special contribution of the Heart Failure Association (HFA) of the ESC. Eur Heart J.

[7] Butler J, Fonarow GC, Zile MR, Lam CS, Roessig L, Schelbert EB (2014). Developing therapies for heart failure with preserved ejection fraction: current state and future directions. JACC Heart Fail.

[8] Roger VL, Weston SA, Redfield MM, Hellermann-Homan JP, Killian J, Yawn BP (2004). Trends in heart failure incidence and survival in a community-based population. JAMA.

[9] van der Heijden AC, Höke U, Thijssen J (2014). Super-responders to cardiac resynchronization therapy remain at risk for ventricular arrhythmias and benefit from defibrillator treatment. Eur J Heart Fail.

[10] Garan AR, Yuzefpolskaya M, Colombo PC (2013). Ventricular arrhythmias and implantable cardioverter-defibrillator therapy in patients with continuous-flow left ventricular assist devices: need for primary prevention?. J Am Coll Cardiol.

[11] Podrid PJ, Fogel RI, Fuchs TT (1992). Ventricular arrhythmia in congestive heart failure. Am J Cardiol.

[12] Boas R, Thune JJ, Pehrson S, Køber L, Nielsen JC, Videbæk L, Haarbo J, Korup E, Bruun NE, Brandes A, Eiskjær H, Thøgersen AM, Philbert BT, Svendsen JH, Dixen U (2021). Prevalence and prognostic association of ventricular arrhythmia in non-ischaemic heart failure patients: results from the DANISH trial. Europace.

[13] Saito K, Kondo Y, Takahashi M, Kitahara H, Nakayama T, Fujimoto Y, Kobayashi Y (2021). Factors that predict ventricular arrhythmias in the late phase after acute myocardial infarction. ESC Heart Fail.

[14] Wilson JR, Schwartz JS, Sutton MS, Ferraro N, Horowitz LN, Reichek N, Josephson ME (1983). Prognosis in severe heart failure: relation to hemodynamic measurements and ventricular ectopic activity. J Am Coll Cardiol.

[15] Teerlink JR, Jalaluddin M, Anderson S, Kukin ML, Eichhorn EJ, Francis G (2000). Ambulatory ventricular arrhythmias in patients with heart failure do not specifically predict an increased risk of sudden death. PROMISE (Prospective Randomized Milrinone Survival Evaluation) Investigators. Circulation.

[16] Lip GY, Heinzel FR, Gaita F, Juanatey JR, Le Heuzey JY, Potpara T (2015). European Heart Rhythm Association/Heart Failure Association joint consensus document on arrhythmias in heart failure, endorsed by the Heart Rhythm Society and the Asia Pacific Heart Rhythm Society. Eur J Heart Fail.

